# Knowledge-based IMRT planning for individual liver cancer patients using a novel specific model

**DOI:** 10.1186/s13014-018-0996-z

**Published:** 2018-03-27

**Authors:** Gang Yu, Yang Li, Ziwei Feng, Cheng Tao, Zuyi Yu, Baosheng Li, Dengwang Li

**Affiliations:** 1 Shandong Province Key Laboratory of Medical Physics and Image Processing Technology, School of Physics and Electronics, Shandong Normal University, No.88, Wenhua East Road, Lixia District, Jinan, Shandong 250014 China; 2 Shandong Medical Imaging and Radiotherapy Engineering Research Center, Department of Radiation Oncology, Shandong Cancer Hospital, Jinan, 250014 People’s Republic of China

**Keywords:** Specific model, Knowledge-based planning, IMRT, Liver cancer

## Abstract

**Background:**

The purpose of this work is to benchmark RapidPlan against clinical plans for liver Intensity-modulated radiotherapy (IMRT) treatment of patients with special anatomical characteristics, and to investigate the prediction capability of the general model (Model-G) versus our specific model (Model-S).

**Methods:**

A library consisting of 60 liver cancer patients with IMRT planning was used to set up two models (Model-S, Model-G), using the RapidPlan knowledge-based planning system. Model-S consisted of 30 patients with special anatomical characteristics where the distance from planning target volume (PTV) to the right kidney was less than three centimeters and Model-G was configurated using all 60 patients in this library. Knowledge-based IMRT plans were created for the evaluation group formed of 13 patients similar to those included in Model-S by Model-G, Model-S and manually (M), named RPG-plans, RPS-plans and M-plans, respectively. The differences in the dose-volume histograms (DVHs) were compared, not only between RP-plans and their respective M-plans, but also between RPG-plans and RPS-plans.

**Results:**

For all 13 patients, RapidPlan could automatically produce clinically acceptable plans. Comparing RP-plans to M-plans, RP-plans improved V_95%_ of PTV and had greater dose sparing in the right kidney. For the normal liver, RPG-plans delivered similar doses, while RPS-plans delivered a higher dose than M-plans. With respect to RapidPlan models, RPS-plans had better conformity index (CI) values and delivered lower doses to the right kidney V_20Gy_ and maximizing point doses to spinal cord, while delivering higher doses to the normal liver.

**Conclusion:**

The study shows that RapidPlan can create high-quality plans, and our specific model can improve the CI of PTV, resulting in more sparing of OAR in IMRT for individual liver cancer patients.

## Background

Liver cancer is the fifth most common type of cancer and the third leading cause of cancer-related death worldwide. Consequently, liver cancer is an issue that needs to be urgently addressed [1]. Intensity-modulated radiotherapy (IMRT), based on computerized treatment plan optimization, permits the delivery of higher therapeutic doses to the target volume, while reducing the impact on adjacent normal tissue [2]. However, each step in the clinical workflow, from contouring to delivery, contains variability and uncertainties that ultimately translate into inconsistencies and inefficiency [3, 4].

A variety of solutions were studied, to reduce the variability present the back and forth between plan creation and approval [5–10]. Among these methods, knowledge-based planning is the most commonly used. It can predict achievable planning target volume (PTV) and DVHs for organs-at-risk (OARs) for prospective patients, utilizing models built using a library consisting of previous plans [11–19]. Chanyavanich et al. [18] used an algorithm based on mutual information to identify similar patients, and then generate new plans for the target cases. Zhu et al. [20] developed an evaluation tool for quantification, that generates DVHs based on organ volumes, as well as distance-to-target histograms (DTH), using a machine learning approach. Wu et al. [16] predicted DVHs of target cases by establishing the overlap volume histogram (OVH). Based on that, Varian developed the Geometry-based Expected Dose (GED) algorithm and build a commercially available knowledge-based planning solution (RapidPlan, Varian Medical Systems, Palo Alto, CA), which semi-automates the treatment planning process. It uses a library of previous plans to build a model that can achieve OAR DVHs prediction range for a new patient, and subsequently guides the volumetric modulated arc therapy (VMAT) or IMRT optimization process using the Eclipse treatment planning system, along the inferior boundary of the DVH-prediction range. Previous work suggested that RapidPlan achieved clinically acceptable plans for different treatment sites [21–27]. To verify whether a model is suboptimal, Hussein et al. [24] suggested that there should be an insignificant effect on resulting plan quality when removing dosimetric outliers from the model training set. Similarly, Delaney et al. [28] found that statistical outliers removed from or added to models (5-10 outliers) had only a marginal impact on plan results. In a study by Tol et al. [27], a model created using 30 plans generated plans that were similar to those in a model based on 60 plans, when their plans were selected arbitrarily from all of 90 plans.

In this study, two models were developed, one general model and one specific model. All the studies mentioned above are based on the general model, which suits a wide variety of patient cases. However, the prediction ability of specific models tailored for specific patient cases, with specific anatomical features, has not been investigated. The aim of this study was (1) to evaluate the accuracy of RapidPlan prediction capability in IMRT (Eclipse, Varian) plans for individual liver cancer patients by using model libraries consisting of different total number of plans, with different similarity; and (2) to investigate the prediction capability of the general model vs. our specific model.

## Methods

### Clinical plans

Liver cancer patients were treated with IMRT from 2015 to 2016, planned based on the Eclipse treatment planning system. For all patients, the IMRT plans were created using 5 non-uniformly distributed coplanar fields (0°, 200°, 240°, 280°, 320°) with the same photon beam setting of DVO, including number and beams energy. The prescribed dose was set as 50 Gy in 25 fractions. All plans were normalized to a mean dose of PTV (and isodose 95% was set to the prescribed dose) in order to make plan comparisons valid. OARs planning goals included maximizing point doses to the spinal cord and their planned at-risk volumes (3-mm expansion) below their tolerance dose levels, while lowering the endpoint dose to normal liver tissue and right kidney as much as possible. The PTV dose-volume constraints and OARs dose constraints are shown in Table 1. All clinical plans were manually optimized by an expert liver dosimetrist and each IMRT plan met the clinical protocol. Patients exhibited high variability in tumor position and size, as well as OAR exposure. All optimization and dose calculations were performed the dose volume optimization (DVO) version 13.5.35 and the anisotropic analytical algorithm (AAA) version 13.5.35 with a calculation grid of 2.5 mm. IMRT planning in Eclipse creates highly conformal dose distributions for liver cancer by continuously optimizing the beam intensity modulation to satisfy the institutional optimization protocol. Eclipse IMRT planning combines intensity modulation and inverse planning to accomplish this goal [27].

**Table 1:** Dose-volume endpoint evaluation

Structures	Acceptable criteria
PTV	V_95%_ > 95%
	D_98%_ > 47.50 Gy
	D_2%_ < 55.02 Gy
Normal liver	Dmean < 23 Gy
	V_30Gy_ < 28%
	V_40Gy_ < 24%
Spinal cord	Dmax < 40 Gy
Right kidney	Dmean < 18 Gy
	V_5Gy_ < 70%
	V_10Gy_ < 55%
	V_15Gy_ < 35%

Abbreviations: *PTV* planning target volume, *Dmean* the mean dose for the normal liver or right kidney, *Dmax* the maximum dose for the spinal cord, *VxGy* volume receiving at least XGy, *D*_*Y*%_ dose delivered to at least Y% of the volume

### Model library and DVH estimation model configuration

The model library consisted of 60 liver cancer patients treated as above. From this library, all 60 patients were selected for Model-G. The average target volume was 147.2 ± 83.6 cm^3^ (range: 15.7-298.3 cm^3^). No other specific criteria were applied. For Model-S, 30 patients were selected, with the distance from PTV to the right kidney of less than 3 cm. The average target volume was 155.69 ± 77.5 cm^3^ (range: 30.4-306 cm^3^). Table 2 shows the volumes of the PTV, liver, L-PTV (normal liver) and right kidney, included in the two models.

**Table 2:** Size of PTV and OAR of patients in Model-G, Model-S and the evaluation group

	Volume				
Group	(cm3)	PTV	Liver	L-PTV	Kidney-R
Model-G	Mean	149.2 ± 83	1580.1 ± 501.3	1452.8 ± 480	172.7 ± 36.4
	Range	15.7-298.3	779.2-2992.2	733.1-2905.9	114.7-280
Model-S	Mean	158.9 ± 78	1539.6 ± 441.7	1430.8 ± 422.4	176.4 ± 37.6
	Range	30.4-306	779.2-2773.6	733.1-2712.1	115-280
EG	Mean	163.1 ± 73.9	1550.3 ± 542.5	1354.0 ± 434.9	184.4 ± 44.1
	Range	24-287.5	822-2896	689.3-2265	122.6-292

Abbreviations: *L-PTV* normal liver structures, *Kidney-R* right kidney structures, *EG* evaluation group

Data were averaged over their respective patients. The range shows the smallest and largest volume deviations

RapidPlan was used to create a knowledge-based model that predicts achievable ranges of DVHs for individual OARs of prospective patients. The model libraries contain all planning CTs, structure sets, and dose distributions of previously treated patients. The model configuration should conceptually consist of multiple kinds of PTV and OAR geometries and alterations in OAR dosimetry, resulting exclusively from geometric alterations.

During the training, the system analyzed the patient anatomy and DVHs in the plans using principal component analysis (PCA), and created the final mathematical DVH estimation model. Then, the results of the model training were verified using statistical presentations of the training set. Regression, residual and DVH-plots help in estimating the quality of the model and finding potential outlier values that differ from the average in the training set [25]. The outliers must be processed, and after that, the plan data are re-extracted and the model is retrained iteratively until the results are acceptable.

An OAR is designated as an outlier when one or more of these metrics lie outside the range of values found in the model. Using this strategy, 14 of 60 patients in Model-G and 6 of 30 patients in Model-S were identified as containing 1 outlier OAR. RapidPlan requires each OAR to be present in at least 20 plans included in the model. We removed statistical outliers from the training set, rather than deleting the whole plan. Table 3 shows the number of structures included in the two models.

**Table 3:** Number of structures included in Model-G and Model-S

Group	PTV	B-P	Liver	L-PTV	Kidney-R	SC	SC-0.3
Model-G	59	57	58	58	53	50	52
Model-S	28	28	28	29	25	27	23

Abbreviations: *B-P* body-PTV, *L-PTV* normal liver structures, *Kidney-R* right kidney structures, *SC* spinal cord, *SC*-0.3 spinal cord’s planning at-risk volumes (3-mm expansion)

### Evaluation group

An evaluation group consisting of 13 previous patients, treated in 2016, was used to test the RapidPlan results. Patients in the evaluation group were not included in the RapidPlan model libraries. The evaluation group was similar to that included in Model-S, where the distance from the right kidney to PTV was less than 3 cm. In the evaluation group, the average target volume was 163.1 ± 73.9 cm^3^ (range: 24-287.5 cm^3^). OARs typically included the liver, L-PTV (normal liver), the spinal-cord and their planned at-risk volumes (3-mm expansion). The volumes of the PTV, liver, L-PTV and right kidney, included in the evaluation group, are shown in Table 2.

The two models were used to generate optimization objectives and automatically optimize treatments for patients in the evaluation group, using the Eclipse treatment planning system. The PTV and OAR objectives are shown in Table 4. Optimization and dose calculation were performed using the Photon Optimization (PO) version 13.5.35 and AAA version 13.5.35. Knowledge-based IMRT plans were created for the evaluation group by Model-G and Model-S, named RPG-plans and RPS-plans, respectively, collectively termed RP-plans. Furthermore, these plans were manually optimized by an expert liver physicist, defined as M-plans.

**Table 4:** The PTV and OAR optimization objectives for evaluation group

Structures	Type	Volume [%]	Dose	Priority
PTV	Upper	0.0	5500 cGy	500
	Upper	2.0	5400 cGy	500
	Lower	100	5000 cGy	500
	Lower	98	5050 cGy	500
Spinal cord	Upper	0.0	3500 cGy	150
Right kidney	Line	Generated	Generated	100
Normal liver	Line	Generated	Generated	100

Abbreviations: *PTV* planning target volume

### Evaluating the performance of RapidPlan

Comparisons of the differences in the DVHs, not only between RP-plans and their respective M-plans, but also between RPG-plans and RPS-plans were performed. RapidPlan results were compared based on target dose coverage and normal tissue sparing. The target dose coverage includes: (1) the homogeneity index (HI) calculated for PTV using [HI = ((D_2%_ - D_98%_)/D_p_) × 100%], where D_p_ = prescribed dose; a lower HI value indicates that the dose coverage is more homogeneous [19]; (2) the conformity index (CI) proposed by Nakamura et al. [29]: CI = TV × PIV/TV_PIV_^2^, where TV = target volume, PIV = prescribed isodose volume, and TV_PIV_ = target volume receiving the prescribed dose. The dose coverage and conformity are better when the value of CI is closer to 1. The normal tissue sparing is based on statistical average doses to normal liver (D_mean_, V_30Gy_ V_30Gy_, V_40Gy_), spinal-cord (D_max_), and right kidney (D_mean_, V_5Gy_, V_10Gy_, V_15Gy_). Paired t-tests were performed to determine significant differences (*p* <  0.05) between RP-plans and their respective M-plans. The target and normal tissue constraints shown in Table 1 were used to compare all patients in the evaluation group.

## Results

**Fig. 1:**
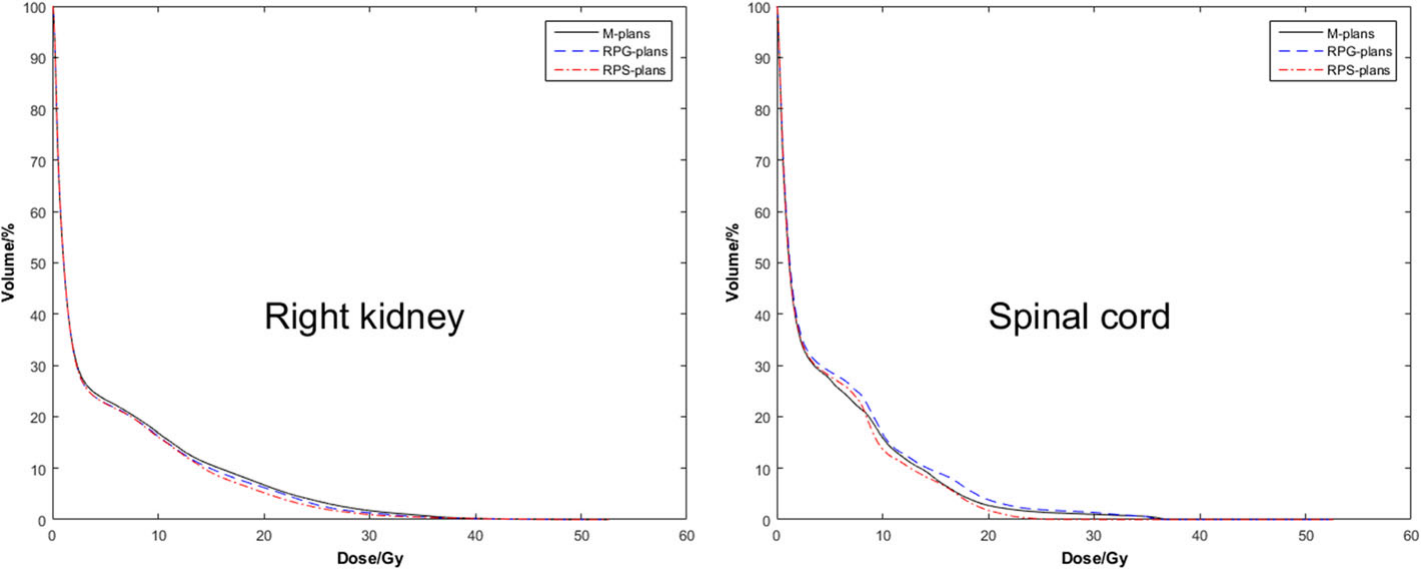
Average DVHs for the M-plans (solid lines), RPG-plans (dashed lines), RPS-plans (dotted lines) for the evaluation group

**Fig. 2:**
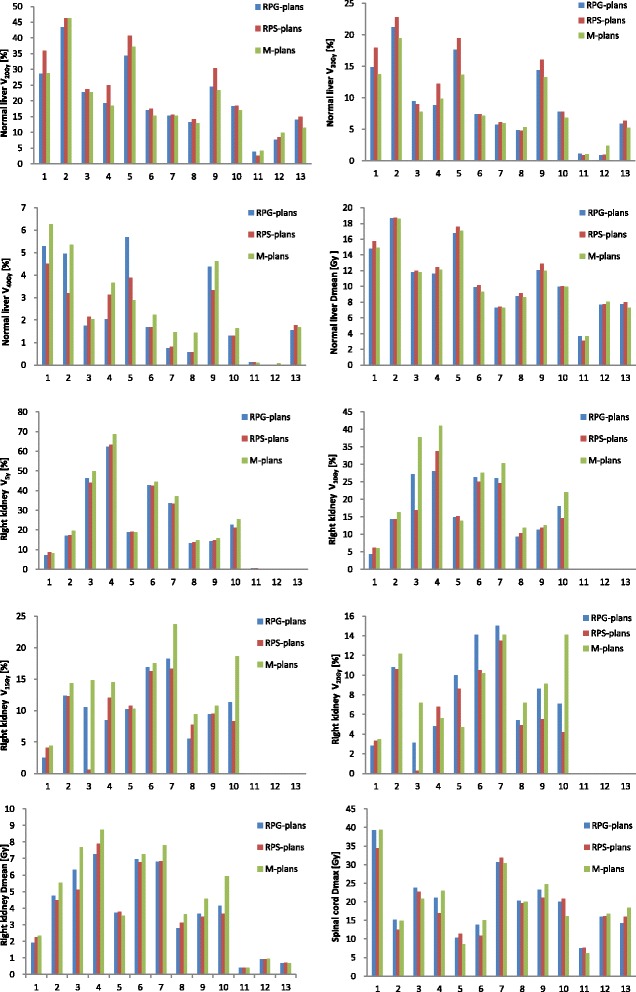
Histograms show the dosimetric values of respective parameters for the RP-plans and the M-plans of all patients in the evaluation group

All knowledge-based plans were deemed to conform with the liver IMRT clinical protocol used at our institution, regarding dose-volume constraints. Table 5 and Fig. 1 summarize the RapidPlan results for the evaluation group, averaged over all patients, whereas Fig. 2 shows results for individual patients.

**Table 5:** Dosimetric comparison of M-plans, RPG-plans and RPS-plans; data shown are the average of their respective parameters for the 13 patients

Structures	M-plans	RPG-plans	RPS-plans	*P*
PTV V_95%_ [%]	97.9 ± 1.1	98.4 ± 0.8	98.5 ± 0.6	i: < 0.05, ii: < 0.05
	[95.3-99.4]	[96.6-99.4]	[97.4-99.4]	iii: NS
PTV D_98%_ [Gy]	50 ± 0.5	50.2 ± 0.5	50.2 ± 0.3	i: NS, ii: NS
	[48.8-50.8]	[49.8-50.9]	[49.7-50.6]	iii: NS
PTV D_2%_ [Gy]	54.8 ± 0.3	54.7 ± 0.2	54.8 ± 0.2	i: NS, ii: NS
	[54-55.1]	[54.4-55]	[54.4-55.1]	iii: NS
PTV HI (%)	9.4 ± 1.4	9.1 ± 1.2	9.1 ± 0.9	i: NS, ii: NS
	[8.4-12.8]	[7.8-11.7]	[7.4-10.9]	iii: NS
PTV CI	1.113 ± 0.037	1.117 ± 0.039	1.102 ± 0.05	i: NS, ii: NS
	[1.06-1.191]	[1.037-1.207]	[1.069-1.177]	iii: < 0.05
Normal liver Dmean [Gy]	10.8 ± 4	10.8 ± 4	11.1 ± 4.2	i: NS, ii: < 0.05
	[3.7-18.6]	[3.7-18.6]	[3.1-18.7]	iii: < 0.05
Normal liver V_20Gy_ [%]	20.1 ± 11.1	20.1 ± 10.3	22.6 ± 12.3	i: NS, ii: < 0.05
	[4.1-46.3]	[3.8-43.4]	[2.6-46.2]	iii: < 0.05
Normal liver V_30Gy_ [%]	8.6 ± 5	9.2 ± 5.9	10.1 ± 6.7	i: NS, ii: < 0.05
	[1.1-19.4]	[0.9-21.2]	[0.9-22.8]	iii: NS
Normal liver V_40Gy_ [%]	2.6 ± 1.8	2.3 ± 2	2 ± 1.4	i: NS, ii: < 0.05
	[0.1-6.3]	[0.0-5.7]	[0.0-4.5]	iii: NS
Spinal cord Dmax [Gy]	19.6 ± 8.4	20.7 ± 8.2	19.0 ± 7.6	i: NS, ii: NS
	[6.2-39.5]	[7.5-39.3]	[7.7-34.4]	iii: < 0.01
Right kidney Dmean [Gy]	4.5 ± 2.8	3.9 ± 2.4	3.8 ± 2.3	i: < 0.01, ii: < 0.01
	[0.4-8.7]	[0.4-7.3]	[0.4-7.9]	iii: NS
Right kidney V_5Gy_ [%]	22.3 ± 20.4	21.5 ± 18.8	21.5 ± 18.6	i: < 0.01, ii: < 0.01
	[0.1-63.4]	[0.2-62.3]	[0.2-63.4]	iii: NS
Right kidney V_10Gy_ [%]	16.9 ± 13.5	13.8 ± 10.3	13.3 ± 10	i: < 0.05, ii: < 0.05
	[0.0-41]	[0.0-28]	[0.0-33.7]	iii: NS
Right kidney V_15Gy_ [%]	10.7 ± 6.7	8.1 ± 5.9	7.6 ± 5.9	i: < 0.01, ii: < 0.05
	[0.0-18.7]	[0.0-18.3]	[0.0-16.6]	iii: NS
Right kidney V_20Gy_ [%]	6.0 ± 4.5	5.6 ± 4.6	4.5 ± 4.0	i: NS, ii: NS
	[0.0-12.8]	[0.0-13.7]	[0.0-12.7]	iii: < 0.05

Abbreviations: *HI* homogeneity index, *CI* conformity index, *RPG-plans* RapidPlan plans using Model-G, *RPS-plans* RapidPlan plans using Model-S, *NS* not significant, *i* M-plans vs. RPG-plans, *ii* M-plans vs. RPS-plans, *iii* RPG-plans vs. RPS-plans. *P* ≤ .05 indicates a statistically significant change

The comparison of RP-plans to M-plans revealed that RP-plans significantly improved V_95%_ of PTV (RPG-plans vs. RPS-plans vs. M-plans: 98.4% vs. 98.4% vs. 97.9%). The D_98%_, D_2%_, HI and CI values of PTV were improved, although most differences were not significant. For the OAR, RP-plans had significantly more sparing of the right kidney (Dmean: RPG-plans vs. RPS-plans vs. M-plans, 3.9 Gy vs. 3.8 Gy vs. 4.5 Gy). For the normal liver, RPG-plans delivered similar doses, while RPS-plans delivered higher doses than those delivered by M-plans (Dmean: RPG-plans vs. RPS-plans vs. M-plans, 10.8 Gy vs. 11.1 Gy vs. 10.8 Gy). No difference was found between RP-plans and M-plans in the maximum dose to the spinal cord.

With respect to RapidPlan models, there are different. For the PTV coverage, the V_95%_, D_98%_, D_2%_, and HI values were very similar, but RPS-plans had better CI values (1.117 vs. 1.102, *p* <  0.05). For OAR doses paring, RPS-plans delivered a lower dose than the maximizing point doses to spinal cord (19.0 Gy vs. 20.7 Gy). Regarding the dose sparing for the right kidney, RPS-plans were better than RPG-plans, especially with V_20Gy_ (4.5% vs. 5.6%, *p* <  0.05), although others were very similar. Figure 1 shows the advantages of RPS-plans in the DVH curves for the right kidney and spinal cord. For normal liver, RPS-plans delivered a higher dose (Dmean: 10.8 Gy vs. 11.1 Gy, *p* <  0.05; V_20Gy_: 20.1% vs. 22.6%, *p* <  0.05), although all RPS-plans were deemed conform with the liver IMRT clinical protocol. However, for certain individual patients, such as patient 11 (Fig. 2), RPG-plans are better for normal liver sparing.

## Discussion

Fogliata et al. [21] tested RapidPlan for the optimization of RapidArc plans, and generated clinically acceptable plans for hepatocellular cancer radiotherapy. This shows that the model is reliable when no special selection criteria are applied to generate the training, i.e. including all cases, for which the only requirement is to be clinically acceptable. However, liver cancer has high variability in tumor size and position, and the general model (Model-G) may have limited accuracy for special patients. Thus, a specific model (Model-S) was established in this study, using plans with specific anatomical features for individual patients. For Model-G, Jol et al. [27] demonstrated that 30 plans were sufficient for building a general model. However, in this study, Model-G consisted of 60 patients, to ensure the variety of the data, whereas Model-S consisted of 30 patients, to guarantee high similarity in the geometry of the region of interest. The scope of the minimum reasonable sample size will be addressed in further studies. Nevertheless, Jim et al. [27] observed that more OAR outliers did not necessarily translate into a worse OAR dose. Hussein et al. [24] showed that there were insignificant effects on resulting plan quality when removing dosimetric outliers from the model training set.

In this study, we evaluated the prediction capability of two models (Model-G & Model-S) in knowledge-based IMRT planning for individual liver cancer patients. Pooled results show that, generally, RapidPlan can improve planning quality and efficiency for liver IMRT, and the prediction ability of the two models with different configurations have a remarkable difference. RP-plans significantly improved the target coverage and the sparing of the right kidney compared to the M-plans. The advantages of some RP-plans compared to the M-plans may be due to challenges in performing an interactive planning of plans that contain many OARs optimally and consistently in a limited number of iterations. Comparison of the two models revealed that Model-S improved the CI and delivered lower dose to the right kidney (V_20Gy_) and spinal cord, while Model-G delivered lower dose to normal liver tissue. This indicates that the RP-plans are sensitive to the configuration of the model library and the anatomical characteristics of the patient that knowledge-based planning is performed on. The degree of similarity of the cases that make up the model library has a significant effect on the predictive capability of the model.

The selection criteria for establishing the specific model in this study was that the distance from PTV to the right kidney should measure less than 3 cm. We expected to better protect the right kidney, while improving the target coverage, and the experimental results show that Model-S achieved substantial gains compared to Model-G. The pooled data (Table 5 and Fig. 2) shows that RPS-plans slightly improved the right kidney sparing. However, for certain individual patients, RPS-plans had a significant advantage when compared to RPG-plans. For example, in patient 3 (Fig. 1), high variability in right kidney sparing was obtained, RPS-plans decreased V_10Gy_, V_15Gy_, V_20Gy_ and Dmean to the right kidney compared to RPG-plans. In patient 11 (Fig. 2), RPG-plans are better for normal liver sparing. From an anatomical point of view, the reason may be that the PTV was small (24 cm3), and located in the inferior segment of the left side of the liver. In addition, it is encouraging that RPS-plans significantly reduced the maximum dose of the spinal cord compared to RPG-plans. This may be due to the fact that the spinal cord has a relatively fixed geometry, adjacent to the kidneys. Therefore, the specific model has great potential in clinical applications for individual patients and we will focus on this.

Some of the findings indicate the different tradeoffs in knowledge-based planning results. According to the pooled results of dosimetry, Model-G is better than the Model-S for normal liver sparing. The following factors may contribute to this: (1) the large size of the liver, only considering the cases with short distance between PTV and the right kidney may not meet the requirements for livers located far away from right kidney; (2) Model-G consisted of more cases, which increases geometric heterogeneity. It is conceivable that further improvements could be made and/or some complex tradeoffs should be addressed. Therefore, some parameters may be adjusted during the optimization process. It is worth noting that a review of plans used for RapidPlan models is still required.

RapidPlan results depend on a variety of factors and the following two are foremost, the geometry of the region of interest and the quality of the plans contained in the model libraries. To ensure the unity of the variables, we built a specific model, which only considers the geometric distance from the PTV to the right kidney, with no restrictions on the other OAR. In addition, one limitation of the study is the small sample size of the evaluation group (*n* = 13). Our study can provide some guidance for clinical applications. Future research will focus on providing optimal allocation of model libraries for individual patients.

## Conclusion

This study shows that RapidPlan can create high quality plans and significantly improve the planning efficiency of IMRT for individual liver cancer patients. Furthermore, these findings demonstrate that the specific model can result in more sparing of OAR, while increasing the conformity index of PTV for liver cancer. Although more systematic studies are needed before a broad clinical application of the proposed methodology, this specific model might be considered as a way to improve the planning quality. Further studies are needed to determine the optimal composition of model libraries, including the relationship between model composition and dosimetry of subsequent plans.

## Abbreviations

AAA: Anisotropic analytical algorithm
CI: Conformity index
DTH: Distance-to-target histograms
DVHs: Dose-volume histograms
DVO: Dose volume optimization
GED: Geometry-based expected dose
HI: Homogeneity index
IMRT: Intensity-modulated radiotherapy
Model-G: General model
Model-S: Specific model
M-plans: Plans were created by manually
OARs: Organs-at-risk
OVH: Overlap volume histogram
PCA: Principal component analysis
PO: Photon optimization
PTV: Planning target volume
RPG-plans: Plans were created by Model-G
RPS-plans: Plans were created by Model-S
VMAT: Volumetric modulated arc therapy
